# The neuroscience of music perception: a narrative review

**DOI:** 10.1055/s-0045-1811233

**Published:** 2025-08-31

**Authors:** Renan Barros Domingues, Luísa Aires Domingues, Victor Rebelo Procaci, José Luiz Pedroso

**Affiliations:** 1 Santa Casa de Misericórdia de São Paulo, Faculdade de Ciências Médicas, São Paulo SP, Brazil.; 2 Senne Liquor Diagnóstico, São Paulo SP, Brazil.; 3 Universidade Federal de São Paulo, Escola Paulista de Medicina, Departamento de Neurologia e Neurocirurgia, São Paulo SP, Brazil.

**Keywords:** Neurosciences, Music, Epilepsy, Hallucinations, Alzheimer Disease

## Abstract

The present review article explores the neuroscience of musical perception, examining the roles of specific brain regions in decoding and interpreting music. Musical perception engages multiple cortical and subcortical areas that work in an integrated manner to process musical elements such as melody, harmony, and rhythm. The paper reviews the current knowledge about the brain circuits involved, as well as pathological conditions that result in abnormalities of musical perception. In addition, the relationship between musical perception and neurological conditions such as epilepsy and Alzheimer's disease is explored. The present review is based on findings from structural and functional neuroimaging studies, neuropsychology, neurophysiology, and clinical research, aiming to show how the brain transforms music sounds into meaningful experiences and addressing pathological conditions in which this complex process may be affected, either in isolation or in association with other forms of neurological impairment.

## INTRODUCTION

Music is an art form and a cultural expression that is rooted in human emotions, thoughts, and traditions. Music transcends language and can evoke a wide range of feelings, from joy and excitement to nostalgia and serenity. While its interpretation varies across cultures and individuals, music is universally recognized as a fundamental part of human experience, helping to connect people and supporting the creation of identity and sense of belonging. 1 2

Humans have an almost universal ability to recognize music, even when exposed to unfamiliar styles, rhythms, or instruments. This innate capacity reflects the brain's specialized networks for processing musical elements, creating a sense of structure that most individuals can understand instinctively. This process is called music perception, which is a complex ability that involves multiple brain areas that work together to recognize and analyze music and evoke emotional and associative responses. 3

Like most brain functions, music perception is influenced by a variety of factors, including genetics and training. Genetic influences may affect the ability to recognize pitch, rhythm, and harmony, leading to variations in musical talent and sensitivity across individuals. 4 Environmental factors, such as early exposure to music and cultural context, also play a significant role in shaping musical abilities, including music perception. 5 Music perception can be impacted by certain rare disorders.

The purpose of this review is to explore the physiological and pathological aspects of music perception, a brain function that is rarely addressed in conventional neurology training.

## METHODS

The present critical review was conducted through a comprehensive literature search in the PubMed/MEDLINE and LILACS electronic medical databases, focusing on peer-reviewed articles published from 2000 to the present. The search strategy employed combinations of the following key terms: *music perception* , *music emotion* , *amusia* , *congenital amusia* , *absolute pitch* , *musicogenic epilepsy* , *musical seizures* , *musical hallucinations* , *music memory* , and *music and Alzheimer Disease* .

During the selection process, the reference lists of initially identified articles were also examined, leading to the inclusion of additional relevant studies not retrieved in the primary search. The review encompassed articles of different methodological designs, including systematic and narrative reviews, clinical studies involving patients with neurological or psychiatric conditions, and experimental studies with healthy volunteers, particularly those employing neuroimaging, electrophysiological methods, or behavioral paradigms to investigate musical processing.

The selected literature was critically analyzed to identify the main findings, controversies, and gaps in knowledge regarding the neural and cognitive mechanisms of music perception, as well as the clinical and topographical correlates of its dysfunctions.

## PHYSIOLOGICAL MECHANISMS OF MUSIC PERCEPTION

**Figure 1:**
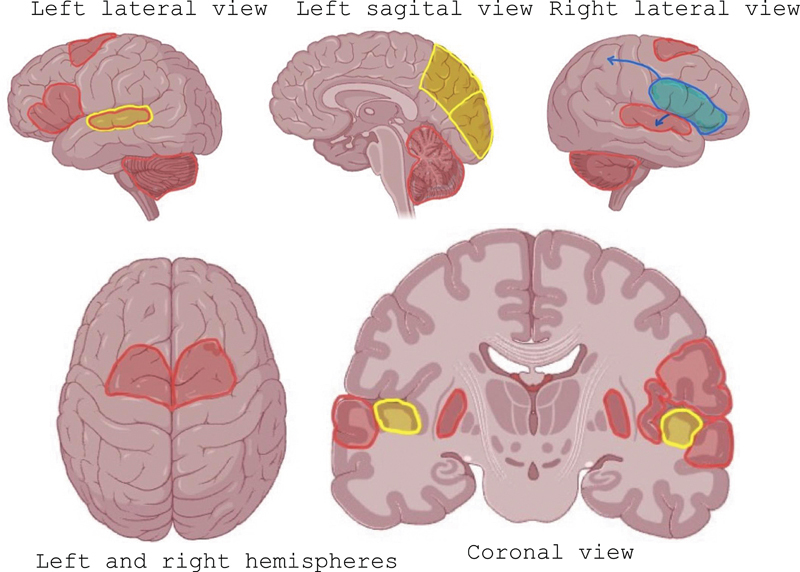
Different brain areas involved in music perception. In yellow, we highlighted the areas associated with melody processing: the auditory cortex, left cuneus, and precuneus. Pitch processing, also in yellow, is primarily localized within the Heschl's gyrus and adjacent areas. In blue, we highlighted the areas associated with harmony processing, the right inferior frontal gyrus, which interacts with the parietal and temporal areas. In red, we highlighted the areas associated with rhythm processing, which involves a bilateral cortico-subcortical network, including the superior temporal cortices, supplementary motor area, putamen, and cerebellum. The left inferior frontal gyrus and insula are implicated in the processing of sequential sounds, which are essential for rhythm. 7 13 24

The brain regions involved in sound processing and music perception are illustrated in Figure 1 . The areas associated with musical perception will be presented, detailing how they enable the brain to perceive music as a distinctive and unique phenomenon.

### Auditory cortices and hemispheric specialization

The auditory information travels from the auditory nerves to the cerebral cortex, reaching the primary auditory cortex. This area is responsible for the initial processing of auditory information. Sound wave frequencies are preserved through a process called tonotopy, traveling from the ear to the brain in an organized manner that maintains their specific pitches. The primary auditory cortex preserves a tonotopic organization with higher pitched sounds being processed in its posterior region and low-pitched sounds being processed in a more anterior region. This forms a map of tones allowing the brain to accurately differentiate specific frequencies. 6

The hemispheric specialization of the auditory cortex involves a division of tasks. The left hemisphere is more involved in the analysis of temporal and sequential aspects of sound, such as rhythm perception and linguistic processing. The right hemisphere specializes in the perception of pitch and melodic aspects, such as intonation, harmony, and timbre. This division explains why right hemisphere lesions can impair the ability to recognize melodies or detect dissonance, while the basic rhythm perception is intact. It is important to note that this hemispheric specialization is not entirely rigid. 7

The secondary auditory cortex areas are involved in the advanced processing of auditory stimuli:

- Brodmann area 42 provides an initial interpretation of musical and speech characteristics;
- The right hemisphere Brodmann area 22 contributes to music processing, particularly in the perception of tonality, harmony, and consonance, which is crucial for distinguishing pleasant (consonant) sounds from unpleasant (dissonant) ones 2;
- The planum temporale (PT) plays a crucial role in speech perception in the left hemisphere, while the right PT is important for melody perception and differentiating the emotional quality of vocal sounds; 8
- The superior temporal gyrus (STS), especially in the right hemisphere, is associated with non-verbal stimuli, including music and emotional voice aspects like tone and intonatio. 9

The different areas of the secondary auditory cortex work in conjunction. The left hemisphere is more involved in processing linguistic and temporal structures, while the right hemisphere is the most important for melody and harmony perception.

### Perception of musical elements

Music perception is a holistic process in which the brain interprets all musical elements—such as melody, rhythm, harmony, and timbre—as a cohesive experience. However, researchers often study them separately for methodological purposes.

#### *Melody*

Melody perception is a complex process involving multiple areas of the brain, particularly in the right hemisphere. The primary auditory cortex performs a basic analysis of tones, while the secondary auditory cortex (including the STS and adjacent areas in the right hemisphere) integrates tones into melodic patterns. The right hemisphere is particularly important for perceiving melodic and harmonic aspects of music. The PT and STS are involved in distinguishing musical from non-musical sounds and recognizing melodic patterns. After initial melody perception, emotional regions of the brain, such as the amygdala and orbitofrontal cortex, are activated, integrating melody recognition with emotion. 10

The understanding of the consonance (harmonious and pleasant sounds) and dissonance (tense and “unresolved” sounds) perception has received contribution from functional neuroimaging studies. Consonant sounds tend to generate higher activity in the secondary auditory cortex and in limbic regions associated with emotion (such as the orbitofrontal cortex), while dissonant sounds more intensely activate regions associated with threatening or uncomfortable stimuli, such as the amygdala. The right hemisphere is the one more involved in distinguishing consonance from dissonance. 11 12

#### *Rhythm*

Rhythm perception depends not only on auditory areas but also on motor areas that anticipate and synchronize rhythmic pulses. Hemispheric specialization also plays an essential role. The left hemisphere is more active in analyzing complex and sequential rhythms while the right hemisphere contributes more to the analysis of slower and irregular rhythmic patterns. 13

- The premotor cortex and the supplementary motor area (SMA) are crucial for rhythm perception and production. Studies show that they not only coordinate motor actions during beat production but are also activated when a person merely listens to a rhythmic sequence, indicating a connection between rhythm listening and motor preparation; 14 15
- The basal ganglia, including the caudate nucleus and putamen, are also fundamental for rhythm perception and production. They play a role in detecting and maintaining a regular pulse, particularly in continuous rhythms or when individuals need to keep an internal tempo without an external cue; 16
- The cerebellum is important for fine motor control and coordination, as well as temporal perception and precise regulation of rhythmic intervals. It helps adjust small variations in beat duration and supports the perception of complex rhythmic patterns; 17
- The prefrontal and superior parietal cortices play roles in attentional control and auditory working memory, essential for keeping rhythm in mind or following a beat while attending to other information. 17

#### *Harmony*

While melody tends to be linear and follows a single line of notes one after another, harmony is multi-layered, providing a background that complements the melody's progression. There are some differences and specificities in the perception of harmony and melody. Harmony perception requires that the brain integrate multiple frequencies at once. This involves greater participation from the right hemisphere, which is more specialized in the spatial and simultaneous integration of sounds. 12

Functional neuroimaging studies show that consonant chords activate the reward system, providing a sense of pleasure, while dissonant sounds activate regions associated with discomfort and alertness. Consonant chords more often activate the reward system, including limbic areas like the nucleus accumbens and the amygdala. Melody also activates emotional areas, but to a lesser extent. Emotional responses to melody tend to relate more to familiarity and melodic progression than to consonance or dissonance. 18 19

Harmony perception also involves the inferior frontal gyrus and inferior parietal regions, which are crucial for aesthetic evaluation and the simultaneous integration of multiple sound elements. Melodic processing generally relies less on aesthetic integration networks in the frontal and parietal areas, and it is more related with the auditory and motor areas, which follow and anticipate rhythm. 20

#### *Integrated processing*

The perception of melody, harmony, timbre, and rhythm occurs simultaneously, creating a cohesive experience, and it is believed that the processing of music involves unconscious mechanisms that group and organize sounds into patterns, working as a probabilistic system, in which the brain makes predictions about what will happen next based on learned patterns.

The brain automatically groups the musical sounds in multiple dimensions:

a) Temporal grouping: an automatic division of music into rhythmic segments, occurring in the auditory cortex and motor areas, such as the premotor cortex, which are activated even without physical movement, suggesting an unconscious rhythmic anticipation. 21
b) Melodic grouping: the brain identifies melodic patterns based on intervals and pitch direction (such as ascending or descending note sequences). This melodic contour recognition occurs automatically in the superior temporal gyrus. 22
c) Harmonic grouping: This unconscious processing is mediated by the secondary auditory cortex and parietal lobe areas, where harmonic combinations are interpreted automatically. 23

These grouping processes generate coherent patterns. This process involves both bottom-up and top-down processing, where sensory input of the sound is integrated and interpreted based on prior knowledge and expectations. Probabilistic mechanisms play a crucial role in this process by predicting the likelihood of certain musical events, such as pitch sequences, rhythmic changes, or harmonic progressions. These predictions are based on statistical regularities learned from exposure to music, allowing the brain to anticipate upcoming events and respond more efficiently. This predictive coding framework suggests that the brain continuously updates its expectations about musical structure, adjusting its predictions when unexpected changes occur, creating a dynamic interplay between expectation and perception in musical experience. 24 25

### *Emotional processing of music*

The main regions related to emotional response to music are: 26

- Amygdala: It is particularly responsive to intense or unexpected sounds, reacting to changes in harmony or dissonance, triggering emotional responses such as joy, excitement, or sadness, according to the music heard and the listener's expectations;
- Hippocampus: It enhances the emotional impact of music when it evokes specific memories. A familiar melody or music tied to a significant moment activates the hippocampus, reinforcing the emotional response;
- Reward system and musical pleasure: The nucleus accumbens and other areas within the reward system (such as the orbitofrontal cortex) are activated in response to enjoyable music. When exposed to music that evokes a sense of pleasure, this system increases dopamine production.

Functional neuroimaging studies have shown different patterns of activation by listening to music in major or minor keys. Music in major keys activates areas responsible for pleasure and reward, such as the nucleus accumbens and orbitofrontal cortex, enhancing feelings of wellbeing. When exposed to music in minor tones, the brain tends to exhibit preferential activation of the amygdala and medial prefrontal cortex, evoking sadness, melancholy, introspection, and sometimes nostalgia. Minor tones are frequently linked to reflective or somber emotional states, as they tend to elicit a sense of emotional depth and complexity. 18 27

## ABNORMALITIES OF MUSIC PERCEPTION

Musical perception abnormalities encompass a range of conditions that affect the ability to process, interpret, or respond to musical stimuli. These abnormalities can result from structural or functional impairments in the brain regions involved in musical processing. Table 1 summarizes the main disorders of musical perception, their topographic correlations, and causes.

**Table 1:** Types of musical perception disorders and their clinical features, topographic correlations, and causes

Disorder	Features	Topographic correlation	Cause
Congenital amusia	Inability to recognize and remember musical melodies are the key phenotypic features	Involvement of connecting fibers in the fasciculi and corpus callosum.	It is an unknown cause, although a genetic influence has been demonstrated in epidemiological studies.
Musical aphasia	Acquired language and music perception involvement	Left temporal lesion	Stroke, brain tumor, frontotemporal dementia
Acquired amusia	Acquired and isolated music perception involvement	Right temporal superior gyrus	Stroke, brain tumor, frontotemporal dementia
Musicogenic epilepsy	Epileptic seizures triggered by music.	Medial and lateral temporal lobes and insula	Mesial temporal sclerosis and other structural brain abnormalities, anti-glutamic acid decarboxylase 65 encephalitis
Musical hallucination	Auditory hallucination related to music	Central or peripheric lesions involving structures of the auditory system	Hearing loss, focal brain lesions, psychiatric disorders, medications
Absolute pitch	Enhanced ability to identify and name pitches without any reference	Higher connectivity between auditory and memory areas of both hemispheres	Genetic and early musical training

### Amusia

The most representative alteration in this spectrum is amusia, a condition characterized by difficulties in pitch recognition (tonal amusia), rhythm perception (rhythmic amusia), or melodic memory (melodic amusia). The term was August Knoblauch in 1888. 28 This condition can be either congenital, present from birth, or acquired, typically developing because of a brain injury. The different types of amusia are described below:

#### *Congenital amusia*

Congenital amusia is caused by structural and functional changes in the auditory cortex, particularly in the superior temporal gyrus. People with congenital amusia typically cannot detect pitch variations and may be insensitive to basic musical structure. It was first characterized by Peretz in 2002 as a condition in which music perception is abnormal despite normal hearing and preserved cognition. 29

A persistent inability to recognize and remember musical melodies are the key phenotypic features. This deficit extends to musical memory and includes difficulties in perceiving pitch and rhythm. The condition is usually noticed during school years when children show an inability to perform musical tasks, such as singing or playing instruments, even percussion ones. Amusia manifests from birth, and daily exposure to music does not improve the condition. 30 31 32

There is no associated hearing loss, brain damage, or intellectual impairment. There are only inconsistent associations with dyslexia and difficulties in spatial orientation. Language acquisition in individuals with amusia is slightly slower in the early stages but later normalizes. Intellectual development in people with amusia is fully within the expected range. 30 31 32 33 Some famous people considered to have high intellectual capacity, including two presidents of United States, Ulysses S. Grant and Theodore Roosevelt, reportedly had amusia. 34

The diagnosis of congenital amusia can be supported by the Montreal Battery of Evaluation of Amusia (MBEA), which is a battery of tests that assesses musical abilities related to six components of musical processing: contour (pitch direction - ascendant and descendant); scale (tonal encoding of a melody); interval (perception of distances between two successive pitches); rhythm (perception of the temporal dimension of a melody); meter (perception of the pulse of a melody); and musical memory (recognition of musical phrases after implicit storage). An adapted version of the MBEA has been used in Brazil. 27

#### *Neurobiological basis*

Reduced connectivity between the left and right auditory cortices has been observed in individuals with amusia, meaning that musical signals are correctly recruited but processed in a separate way in both hemispheres, creating a dual signal stream which is not jointly analyzed in the brain.

Neuroimaging studies show that people with amusia have higher diffusivity indices in the corpus callosum and in the right occipital fasciculus. In amusic individuals, the axial diffusivity in these structures negatively correlates with musical performance scores. These findings support the recognition of congenital amusia as a disconnection syndrome, as the corpus callosum and occipital fasciculus are structures involved in connecting different cortical areas. 35 Morphological studies have revealed abnormalities in the volume of white and gray matter in the right inferior frontal gyrus and the right superior temporal gyrus in the brains of people with amusia. 36

Magnetoencephalography studies have demonstrated reduced intrinsic connectivity in the auditory cortex of both hemispheres and reduced right frontotemporal feedback connectivity in individuals with amusia compared with controls. 32 One study tested the response to harmonic structures in 16 individuals with amusia compared with 16 controls. The study found a similar reaction in the initial phase of musical detection. Differences appeared in the later integration phase, precluding hierarchical processing of music. The rupture of interhemispheric fibers was suggested as the cause of hemispheric disconnection. 37

It is important to emphasize that these findings are mostly derived from small cohort studies, and replication in larger populations remains limited.

#### *Genetic aspects*

Studies on the genetic role in amusia have primarily been conducted in the context of classical Mendelian genetics. There is an increased prevalence of amusia within families. 37 Monozygotic twins are significantly more likely to share amusia than dizygotic twins. 38 The genetic impact appears more pronounced in deficits related to pitch and melody recognition, while rhythm recognition relies more on environmental factors. 38 A single study indicated a possible association of amusia with a specific mutation, the deletion of the 22q11.2 chromosome region. This same deletion has been described in psychiatric diagnoses, but in the same study, amusia was not associated with psychiatric disorders, suggesting an independent role of this gene in the determination of amusia and psychiatric disorders. 37 It is important to note that most studies addressing the genetic basis of congenital amusia are observational in nature and involve limited sample sizes, which may restrict the generalizability of their findings. Also, although these findings are of interest in understanding the neurobiological basis of amusia, their clinical utility remains limited at present since most studies are exploratory, involve small or specific populations, and lack replication in broader cohorts. Therefore, their use in clinical practice is not currently supported.

### Acquired disorders

#### *Acquired amusia*

Musical aphasia is a specific condition in which both language and music are affected due to brain damage, usually in the left hemisphere, particularly in the Broca's or Wernicke's area. Musical aphasia involves deficits in processing rhythm and melodic structures, impairing musical understanding similarly to how language is affected. It mainly impacts rhythm perception and melodic structure, as the areas responsible for language, usually in the left hemisphere, also play a role in rhythm and structural processing in music. 39

The most well-known case of amusic aphasia occurred with Maurice Ravel (1875–1937), the renowned French composer, conductor, and pianist. In 1927, Ravel began showing symptoms of dementia, with memory and language impairments, ideomotor apraxia, and amusia, which, more than his other deficits, prevented him from continuing his musical activities. 40

#### *Musical aphasia*

Acquired amusia occurs when a person loses the ability to perceive or produce music accurately due to brain injury, without impairing language. Unlike musical aphasia, acquired amusia can occur after isolated lesions in the right hemisphere, which is essential for processing musical characteristics, such as tonality, timbre, and tonal structure. The main causes are right hemisphere stroke affecting the superior temporal gyrus and adjacent areas, frontotemporal dementia affecting the right temporal lobe, and traumatic brain injury. 39

### Absolute pitch

Absolute pitch is a form of musical perception abnormality opposite to amusia, as it is characterized by the rare ability to identify and name pitches without any reference. Neurofunctional and genetic studies have investigated the mechanisms and hereditary bases of this ability.

#### *Neural correlates*

Functional magnetic resonance imaging (fMRI) and positron emission tomography (PET) studies show that absolute pitch is associated with greater activation in the superior temporal gyrus, particularly in the primary auditory area and adjacent regions. 41

There is evidence that individuals with absolute pitch exhibit higher connectivity between the hemispheres, especially through the corpus callosum, facilitating communication between auditory and memory areas. This increased connectivity may help explain why these individuals can automatically associate specific sound frequencies with stored musical pitch representations in memory. 42

Another finding from imaging studies is a larger volume of gray matter in the auditory areas of the left hemisphere in absolute pitch. Structural changes, associated with early training in genetically predisposed individuals, may be related to the ability to identify musical notes with above-average precision. 38 These conclusions are mostly based on cross-sectional imaging studies with selected groups of musicians.

#### *Genetic and environmental factors*

Genetic studies on absolute pitch indicate a significant hereditary influence. The prevalence of absolute pitch is much higher in families with a musical background. Moreover, monozygotic twins have a significantly higher concordance for absolute pitch compared with dizygotic twins, indicating a genetic component. 43

Among candidate genes, *AVPR1A* , located on chromosome 12, is one of the most studied. This gene is associated with traits such as musical memory and tuning ability, and its expression is considered a potential influencer of absolute pitch. 44 Whole-genome expression studies also identified other genes or chromosome regions associated with musical aptitude: *SLC6A4* (17q11.2), *GALM* (2p22), *PCDH7* (4p15.1), *GATA2* (3q21.3), *FOXP2* (implicated in auditory processing), *COMT* (catechol-O-methyltransferase), *SRGAP2* (involved in neural connectivity and synaptic development), and possibly other genes implicated in neural plasticity and memory. 45 Although genetic associations have been explored in the context of absolute pitch, their current clinical applicability is limited. These findings are based on studies with small sample sizes and often lack replication, which restricts their interpretative value and prevents their translation into clinical or diagnostic use.

The development of absolute pitch also appears to depend on environment and early training. Children who begin musical training before the age of 7 are more likely to develop absolute pitch. 46 While early musical training increases the chances of developing absolute pitch, experiments show that adults can also learn and improve their pitch perception, sustainably enhancing their ability to correctly identify pitches. 47

## MUSIC, EPILEPSY, AND MUSICAL SEIZURES

There is a complex relationship between music and epilepsy, including musicogenic epilepsy, in which music acts as a trigger for epileptic seizures. Additionally, the perception of music can also be an auditory manifestation during focal seizures. 48 Another aspect that has been explored is whether passive exposition to music, due to its effects on brain wave patterns, might have a therapeutic effect on epilepsy. Although some studies have shown potentially favorable results, there is limited and low-quality evidence on the antiepileptic effect of music exposition. 49

### Musicogenic epilepsy

This term was first used by Critchley in 1937 to describe focal-onset seizures, with or without altered consciousness, following exposure to music. Music is a rare trigger of seizures, and musical stimuli does not necessarily need to be heard; sometimes, merely thinking about a piece of music can provoke seizures. The latency between the stimulus and the seizure varies from seconds to several minutes. Some patients develop seizures upon hearing a specific musical piece while others report seizures triggered by a particular musical genre, instrument, or composer. Some patients also report that specific timbres cause seizures. 50

Functional imaging studies have shown fronto-occipital and fronto-orbital activation at the onset of the ictus, possibly reflecting an activation of emotions and memories. Invasive electroencephalographic recordings have shown activity in the right medial temporal lobe, extending to the lateral temporal lobe, Heschl's gyrus, the insula, and areas of the frontal lobe. 51

It is unclear whether music triggers seizures by directly affecting auditory cortex brain activity or if the emotional and cognitive components evoked by listening to music initiate the seizure. 48 Although the precise mechanism by which music acts as a trigger is not yet fully understood, a complex interaction between auditory processing circuits and the brain's emotional networks in surely involved. 52 53 The current evidence is based primarily on case reports and small case series.

In recent years, some cases of musicogenic epilepsy have been associated with the presence of autoantibodies against glutamic acid decarboxylase 65 (GAD) with or without associated limbic encephalitis. The use of immunosuppressive medication has been associated with improvement in some of these reported cases. 54 55

### Musical seizures as epileptic manifestations

Musical seizures are those in which the symptoms are related to the perception of music and they can have different forms, including positive phenomena such as musical hallucinations, intense desire for music, ictal singing, whistling, and murmuring; or negative phenomena such as transient aprosody or amusia. 49 When causing positive phenomena, the manifestation is referred to as musical hallucination. Although epilepsy is one of the causes of musical hallucination, there are other more frequent etiologies, as will be discussed in the following section. 56 Musical hallucinations in the context of epilepsy can be divided into three subtypes, comprising ictal, post-ictal, and inter-ictal phenomena. EEG and fMRI studies show that epileptic musical hallucinations arise from within the auditory network. 56

Other manifestations of musical seizures are even rarer than musical hallucinations. Ictal singing localization may vary, with some patients presenting frontal lobe and others temporal lobe onset. Singing can represent a form of automatism of a learned motor pattern. There are few reported cases of ictal whistling. The lateralization is not precisely defined, but the location is generally in the temporal lobe. 57

## MUSICAL HALLUCINATIONS

### Phenomenology and epidemiology

Musical hallucinations are a particular type of auditory hallucination in which the patient hears music, being aware of the hallucinatory nature of the perception. Unlike other auditory hallucinations, such as voices, these hallucinations involve well-structured and familiar music. Evidence in this area is heterogeneous, with some findings based on retrospective case reviews and others derived from imaging studies in individual patients.

Musical hallucinations predominantly affect elderly individuals with hearing loss. 58 Despite their musical content, these hallucinations can be very disturbing. More than two thirds of published cases were in women, and in nearly one third, there was a structural brain lesion.

### Etiologies of musical hallucinations

#### *Hearing loss*

Hearing loss is the most frequent cause of musical hallucinations. It was suggested that patients with inner ear lesions may develop a hyperactive state of the ear. 59 Another possible explanation is that due to the absence of external stimuli the brain creates internal sounds, leading to the perception of non-existent melodies. This phenomenon is comparable to what occurs in people with vision loss, who may experience visual hallucinations, known as Charles Bonnet syndrome. Music hallucinations in patients with hearing loss have been designated as auditory Charles Bonnet syndrome. 60 Treating the hearing impairment can yield significant improvement of music hallucinations. Coping strategies (e.g., more acoustic stimulation) are frequently helpful. Pharmacological treatment can also be successful, with antidepressants being possibly the more helpful drug category for this condition. 61

#### *Psychiatric disorders*

Musical hallucinations can occur in patients with psychiatric disorders, such as schizophrenia, bipolar disorder, and severe depression. In these contexts, hallucinations may occur as part of a broader symptom profile, often accompanied by other types of auditory hallucinations, such as voices. The music in these cases may have emotional significance or be associated with specific memories, which intensifies the hallucinatory experience. 62

#### *Medication and substance use*

The use of certain medications, such as antidepressants, antipsychotics, and benzodiazepines, can trigger musical hallucinations in some individuals. The cause of the association between these substances and musical hallucinations is unknown. These rare reports generally involve individuals over 60 years of age. Interestingly, recreational use of hallucinogenic substances has not been specifically associated with musical hallucinations, possibly due to the more severe nature of the hallucinatory states they induce. 62

#### *Brain lesions*

Lesions in brain regions involved in auditory and memory processing can lead to musical hallucinations. In most described cases, the lesion was in the right hemisphere. Most brain lesions were vascular or tumoral. 58 63 64

## MUSICAL MEMORY AND COGNITION

The relationship between musical perception and other cognitive processes is still not fully understood. The situations presented below suggest that musical perception, in both its cognitive and emotional aspects, may have singular aspects when compared with other types of information processing.

### Role of the hippocampus

The role of the hippocampus in musical memory is still under debate. Some reports on professional and amateur musicians with amnesic syndromes have shown a surprising preservation of musical memory despite extensive bilateral damage to the medial temporal lobe structures, including the hippocampus. However, these findings could reflect the effects of lifelong musical training. 64

A more recent report described a patient with almost no prior musical training who suffered bilateral hippocampal lesions, with no damage to other brain regions, resulting in a severe amnesic syndrome with difficulties in recognizing visual and verbal information. Even so, this patient maintained musical memory integrity, preserving perception of tones, melodic contours, intervals, rhythm, and meter. This report suggests an independence of musical memory and recognition networks. 65

Functional neuroimaging studies support this hypothesis, showing that the consolidation of melodic information may occur independently of the hippocampus, unlike memory for non-musical content. 66 Other studies demonstrate different hippocampal recruitment during stimuli involving musical recognition memory compared with verbal memory stimuli. 67 Clinical and neuroimaging data support the idea that musical recognition memory is a cognitive ability that operates largely independently of networks involved in other memory domains, potentially involving specific brain areas or even other regions within the hippocampus. 68

### Musical memory in Alzheimer's disease

Several studies have shown that patients with Alzheimer's disease (AD) may preserve the ability to play or sing music learned before the onset of dementia, despite severe dementia symptoms, even no longer remembering the music's title or the composer's name. 68 69 Other studies have sought to understand the mechanisms responsible for preserving musical memories in AD patients. These studies have shown that musical episodic memory is as affected as verbal episodic memory in AD patients; however, semantic musical memory is disproportionately preserved. These results are derived from studies involving small cohorts of patients with AD but consistently shown that, from the early stages, there are deficits in episodic musical memory but with largely preserved semantic musical memory. 70 71 72

Structural and functional neuroimaging studies have helped to explain these differences, showing that brain regions involved in encoding long-term musical memory and the degeneration pattern in AD, showing no overlap between more degenerated areas and those of long-term musical memory encoding. The most involved areas in long-term musical memory were the SMA and cingulate gyrus, which are minimally damaged in AD patients. 73 Other studies showed that listening to long-known music generates extensive activation in prefrontal regions, emotion-related areas, motor, auditory, and subcortical regions (cerebellum, putamen, limbic structures). Some of these regions are relatively preserved in AD, at least in the first stages of the disease. In contrast, listening to recently known music generates much less extensive activation of these areas, providing clues about why long-term musical memory, but not short-term musical memory, is relatively preserved in AD. 74 75

### Opportunities for music-based therapy

Music-based interventions, particularly neurologic music therapy (NMT), have emerged as promising tools in the field of neurorehabilitation. Neurologic music therapy utilizes rhythm, melody, and harmony to engage neural networks involved in motor coordination, cognitive processing, and speech production. Among its various applications, music therapy has shown relevance in the management of AD, as it serves as a non-pharmacological adjunct aimed at enhancing quality of life and alleviating neuropsychiatric symptoms. A growing body of evidence suggests that musical engagement can improve mood, reduce agitation, and promote social interaction in individuals with AD. Additionally, musical activities have demonstrated potential benefits in domains such as language function and autobiographical memory retrieval—areas commonly affected by the disease. 76

The therapeutic value of music in AD is supported by neurobiological findings, indicating that music activates brain regions—such as the medial prefrontal cortex and parts of the limbic system—that are among the last to undergo degeneration, offering a neuroanatomical basis for its integration into cognitive rehabilitation. Furthermore, music therapy may induce neuroplastic changes, fostering functional reorganization and the maintenance of residual cognitive abilities. A 2023 systematic review evaluating the effects of music therapy in AD reported that seven out of the eight studies included found significant cognitive improvements, with active musical interventions outperforming passive listening in enhancing orientation, language, memory, and emotional wellbeing. 77 Notably, interventions that incorporated music aligned with patients' personal preferences yielded superior outcomes. 77 Despite these promising results, further research with standardized protocols and validated outcome measures is essential to consolidate the role of music therapy in clinical practice and facilitate its broader adoption in dementia care.

In conclusion, music perception is a complex neurocognitive function that engages widespread and interconnected brain networks, integrating sensory, emotional, and cognitive processes. 78 While considerable progress has been made in understanding its neural substrates and clinical manifestations, several challenges remain. Disorders such as amusia, musicogenic epilepsy, and musical hallucinations are still underrecognized in clinical practice, partly due to limited coverage in neurology and psychiatry training. Although some validated tools for assessing conditions like congenital amusia exist, their use in educational and clinical settings is minimal.

The present review aims to contribute to addressing this gap by providing a structured synthesis of current knowledge on music perception and its disturbances, along with their anatomical and functional correlates. We also highlight areas of uncertainty, including conflicting findings on the neural basis of emotional responses to music and the limited reproducibility of genetic associations, such as those involving absolute pitch. Furthermore, we propose key directions for future research, including the development of standardized diagnostic instruments and prospective studies—particularly focused on musical memory in neurodegenerative diseases like AD, in which music-based interventions show therapeutic potential.

By bridging neuroscience, clinical observation, and rehabilitation, music emerges not only as an object of scientific inquiry but also as a potential tool for enhancing diagnostic insight and improving patient care. A deeper integration of this knowledge into medical education and practice may promote a more comprehensive and humanized approach to neurological and psychiatric disorders.
